# Alternatives to neonicotinoid insecticides for pest control: case studies in agriculture and forestry

**DOI:** 10.1007/s11356-014-3628-7

**Published:** 2014-10-03

**Authors:** Lorenzo Furlan, David Kreutzweiser

**Affiliations:** 1 Veneto Agricoltura, Legnaro, PD Italy; 2 Canadian Forest Service, Natural Resources Canada, 1219 Queen Street East, Sault Ste Marie, ON P6A 2E5 Canada

**Keywords:** Neonicotinoid, Integrated pest management, Agriculture, Maize pests, Forestry

## Abstract

Neonicotinoid insecticides are widely used for control of insect pests around the world and are especially pervasive in agricultural pest management. There is a growing body of evidence indicating that the broad-scale and prophylactic uses of neonicotinoids pose serious risks of harm to beneficial organisms and their ecological function. This provides the impetus for exploring alternatives to neonicotinoid insecticides for controlling insect pests. We draw from examples of alternative pest control options in Italian maize production and Canadian forestry to illustrate the principles of applying alternatives to neonicotinoids under an integrated pest management (IPM) strategy. An IPM approach considers all relevant and available information to make informed management decisions, providing pest control options based on actual need. We explore the benefits and challenges of several options for management of three insect pests in maize crops and an invasive insect pest in forests, including diversifying crop rotations, altering the timing of planting, tillage and irrigation, using less sensitive crops in infested areas, applying biological control agents, and turning to alternative reduced risk insecticides. Continued research into alternatives is warranted, but equally pressing is the need for information transfer and training for farmers and pest managers and the need for policies and regulations to encourage the adoption of IPM strategies and their alternative pest control options.

## Introduction

Systemic neonicotinoid insecticides are used to protect a wide variety of crops. Based on their efficacy to control many insect pests and their systemic activity, they are used extensively in agriculture so that by 2008, neonicotinoids accounted for one quarter of the global insecticide market (Jeschke et al. 2011), and this rate is increasing (Simon-Delso et al. 2014). The extensive use of neonicotinoids in agriculture has undoubtedly met technical and commercial goals, i.e. simplification of agricultural systems and large pesticide applications for pest prevention to maximize efficiencies and profits. However, increasing evidence indicates that this large-scale use results in high broad-spectrum insecticidal activity of the neonicotinoids even at very low dosages, and this has led to serious risk of environmental impact (Henry et al. 2012; Goulson 2013; van der Sluijs et al. 2013, 2014; Whitehorn et al. 2012). The large-scale, often prophylactic use (Goulson 2013) of neonicotinoid insecticides contrasts with the main principle of an integrated pest management (IPM) approach which includes an assessment of economically important pest populations in order to determine if an insecticide treatment is required. The principles of IPM, derived from dozens of years of field experiments and scientific research (Baur et al. 2011), are summarized and made compulsory in the European Union by Directive 2009/128/CE. For an agricultural setting, the procedure is the following:

1. Before taking any decision on pest control, harmful organisms must be monitored by adequate methods and tools, where available; tools should include observations in the field as well as scientifically sound warning, forecasting, and early diagnosis systems;
2. Treatments may then be carried out only where and when the assessment has found that levels are above predetermined economic thresholds for crop protection;
3. If economic thresholds are exceeded, agronomic solutions, mainly rotation, should be considered to avoid damage to maize crops including the interference of newly established pest populations with tillage timing and other modifications, choice and modification of sowing dates, and alterations of rotation sequences;
4. If economic thresholds are exceeded and no agronomic solutions are available, biological control or physical treatment or any other non-chemical pest control method should be considered as a replacement for chemical treatment;
5. If economic thresholds are exceeded and no agronomic solutions, biological control or physical treatments or any other non-chemical pest control methods are available, chemical treatments should be selected among those that pose the lowest risk to environment and human health, and they should be used in a way that minimizes the risk of pest resistance by limiting their use over space and time.

In order to show that alternatives to neonicotinoids for pest control are available and can be feasible, two case studies will be described: (i) treatment of maize crops, in which it was shown that there was a link between neonicotinoids and negative effects on honeybees (Girolami et al. 2012) and (ii) treatment of trees to control an invasive insect pest. The agricultural case study is significant because it concerns cultivation and pest control methods made on large land bases in Italy (thousands of hectares spanning a 25-year period (Furlan 1989; Furlan et al. 2002, 2007b, 2009a, 2011; Ferro and Furlan 2012)) with potential for side effects on the environment. The forestry case study is significant because it presents a unique pest problem in Canada with environmental issues and solutions of its own.

## Case studies of alternative pest management in maize

By 2010, neonicotinoids accounted for 27 % of the world’s total insecticide use (Casida and Durkin 2013), and their application to pest management in maize is among the highest use of the insecticides in agriculture. For example, over 18 million ha of maize (corn) was treated with a neonicotinoid insecticide between 2009 and 2011 in the USA (Brassard 2012). This included over 810 t of clothianidin and 570 t of thiamethoxam applied in 1 year in the USA, most of it in maize crops (Simon-Delso et al. 2014). Production of maize for food, feed, and biofuel is the single largest use of arable land in the USA, and almost all seeds used in maize production are coated with neonicotinoid insecticides (USDA-NASS 2013). Maize production in the European Union is about 14 million ha per year, with France, Romania, Germany, Hungary, and Italy each producing more than 1 million ha per year (Meissle et al. 2010). Neonicotinoid insecticides are applied to maize crops primarily by seed coating and are designed to protect maize seeds, seedlings, and young plants in the early growing season. The increasing use of neonicotinoids, including the use in maize, has been implicated in significant environmental exposure and impacts, including bee disorders and colony collapse, thereby affecting pollination and other ecological services (Goulson 2013; van der Sluijs et al. 2013, 2014; Bonmatin et al. 2014; Chagnon et al. 2014; Pisa et al. 2014).

The first way of reducing insecticide use in Europe in general, and neonicotinoids in particular, is the proper implementation of the IPM strategies proposed by the European Directive 128/2009/EC on the Sustainable Use of Pesticides. This Directive made it compulsory to apply IPM to all crops in the European Union since January 2014. Although IPM strategies are commonly used on plantations such as orchards and vineyards (Baur et al. 2011), they have not been widely introduced for maize and other arable crops in Europe (Furlan et al. 2013). As arable farming often has limited resources in terms of income, labour, and technology, a special effort is needed to ensure that the directive is successful. This means that if IPM is to be introduced for arable crops, there is a need for (a) low-cost strategies, (b) time-effective tools, and (c) economically and environmentally sustainable pesticides or other pest control methods. One way to achieve these goals is to initiate a modern advisory system that can provide online information on crop treatment options and explain technical criteria. This has been demonstrated in Italy by the new *Bollettino delle Colture Erbacee* (“Annual Crops Bulletin”) (http://www.venetoagricoltura.org/subindex.php?IDSX=120). This advisory bulletin is based on a low-cost area-wide pest and disease monitoring system that establishes when and where pest populations pose an economic risk to arable land. Where the risk actually occurs, it advises how the field evaluation should be carried out. Area-wide monitoring is low-cost since it is based on: (a) pheromone traps, which are user-friendly and inexpensive; (b) pest population models using meteorological information (e.g. the Black Cutworm Monitoring and Forecasting programme (Furlan et al. 2001c) and the Davis model for Western corn rootworm egg hatching, Davis et al. 1996); (c) spatial analysis based on GIS mapping (e.g. geostatistics, De Luigi et al. 2011); and (d) agronomic information from a number of areas. In order to ensure that IPM can be applied to arable crops reliably and affordably, the monitoring and assessment must be conducted at both regional and local farm levels where needed.

At the local farm level, the monitoring procedure requires on-the-ground samples to be taken when areas at risk of significant crop damage from a given insect are identified at regional levels (Furlan et al. 2013). Monitoring crop development may also reveal different susceptibility levels and therefore methods of intervention must be adjusted accordingly. Farmers and other practitioners are informed in a timely manner about these issues and trained in how to use the information correctly in a successful IPM plan where production costs are competitive and environmental impacts are limited. The following is a brief description of some IPM options for managing some common insect pests on maize crops in Italy (and applicable to other parts of Europe) without relying on the prophylactic use of neonicotinoids.

### Controlling wireworms (*Agriotes* spp.)

Long-term data suggest that the majority of maize farmland in Italy does not need to be protected with insecticides at sowing (Furlan 1989; Furlan et al. 2002, 2007b, 2009a, 2011, 2014; Ferro and Furlan 2012). Indeed, the percentage of land with high populations of wireworms (a key soil pest in maize farmland) is often very low (e.g. less than 5 % in the Veneto region (Furlan 1989; Furlan et al. 2002, 2007b, 2009a, 2011; Ferro and Furlan 2012), an area with large-scale maize production). At the European level, similar results are coming from the European project PURE (VII Framework). After the first 3 years of monitoring, no significant wireworm damage in the experimental fields of France, Hungary, Slovenia, Germany, and other Italian regions was detected (Furlan, unpublished data). Hundreds of plots have been examined in studies from Italy, and in the large majority of the experiments, there were no statistically significant differences, in terms of yield and crop stand, between maize treated with neonicotinoids and non-treated plots because of low wireworm damage and/or the compensation capacity of the crops (Balconi et al. 2011; Boicelli 2007; Ferro and Furlan 2012; Furlan et al. 2002, 2007b, 2009a, 2011).

These data demonstrate that insecticides are often not needed and may not always contribute effectively to yield gain (Goulson 2013). In these situations, low pest populations determined by monitoring and field assessments may provide information for successful IPM implementation. Because of this general low-risk level, a crop insurance programme where growers may purchase insurance, instead of soil insecticides, to provide financial compensation when yield losses can be attributed to pests would be more feasible than prophylactic protection. The total cost of damage to maize (need of re-sowing and loss of yield due to delayed sowing or reduced stand) is often lower than the total cost of the prophylactic protection of all planted fields (Furlan et al. 2014), and this does not include any consideration of environmental side effects of neonicotinoids (van der Sluijs et al. 2014).

#### Accurate wireworm population monitoring and damage prediction

An effective and sustainable maize production strategy is to plant sensitive crops in areas free of harmful wireworm populations. Currently, some wireworm population levels can be predicted reliably and cost effectively with pheromone traps (Furlan et al. 2001a; Gomboc et al. 2001; Karabatsas et al. 2001; Tóth et al. 2001, 2003), which are suitable for monitoring all of Europe’s main *Agriotes* species (*Agriotes sordidus* Illiger, *Agriotes brevis* Candèze, *Agriotes lineatus* L., *Agriotes sputator* L., *Agriotes obscurus* L., *Agriotes rufipalpis* Brullè, *Agriotes proximus* Schwarz, *Agriotes litigiosus* Rossi, and *Agriotes ustulatus* Schäller). In the last few years, research has provided useful information about the biological significance of pheromone trap catches and has demonstrated their range of attraction (Sufyan et al. 2011). Captured adults (click beetles) in pheromone traps may be correlated with the presence of larvae of the same species in soils, at least for the three main species of southern Europe, namely *A. sordidus* Illiger, *A. brevis* Candèze, and *A. ustulatus* Schäller (Burgio et al. 2005, 2012; Furlan et al. 2001b, 2007a; Pozzati et al. 2006). However, this relationship is less certain for other important European species, such as *A. obscurus* L., *A. lineatus* L., and *A. sputator* L. (Benefer et al. 2012; Blackshaw and Hicks 2013). Spatial models (e.g. geostatistical analyses) are available in Italy, providing predictions of *Agriotes* population dynamics at different spatial scales (i.e. large farms, provinces) which are then interfaced with agronomic and geographic variables, leading to improved analysis of risk and optimization of monitoring costs (Burgio et al. 2005).

The information obtained by pheromone trap monitoring can improve the prediction of population levels and the actual risk of crop damage based on the evaluation of a field’s agronomic and climatic characteristics along with the biological and ecological information of each species (Furlan 1996, 1998, 2004); Masler 1982; Rusek 1972 *;* Kosmacevskij 1955. The two main risk factors are (i) more than 5 % organic matter content of the soil (Furlan 1989, 2005, unpublished data; Furlan et al. 2011) and (ii) continuous plant cover of the soil with meadow or double crops (such as barley and soybean, ryegrass and maize, etc.) in the two previous years (Furlan 1989, 2005, unpublished data; Furlan and Talon 1997; Furlan et al. 2011). If no agronomic risk factors are present, no treatments are needed. When pheromone traps have detected high beetle population densities and/or agronomic risk factors are present, bait traps for larvae (Chabert and Blot 1992; Parker 1994, 1996; Parker et al. 1994) can then be used to pinpoint the areas with wireworm populations that exceed the economic threshold. However, each *Agriotes* species responded differently to bait traps, and consequently, the thresholds for each species must be assessed separately (Furlan 2011). Therefore, species identification is important, and although polymerase chain reaction (PCR) and DNA sequencing are currently available to identify species (Staudacher et al. 2010), other more practical and feasible identification methods should be developed for each region. Data from maize farms in Italy over the last 20 years have enabled researchers to establish that there is a close correlation between the number of larvae per square metre, or between the average number of larvae per bait trap, and the number of maize plants damaged by *A. brevis*, *A. sordidus*, and *A. ustulatus* (Furlan 2014). When wireworm populations are above threshold values, agronomic and biological treatment options should be considered before resorting to chemical treatments.

#### Agronomic strategies for controlling wireworm populations

Crop rotation, food resources, climatic and agronomic conditions (mainly organic matter content), as well as other soil characteristics are the main factors that influence larval population densities (Furlan 2005). Generally, the vast majority of non-sensitive or low-sensitive crops (e.g. soybean) can be planted in identified infested fields, while the remaining cultivated soils can be planted with another sensitive crop, including maize (Furlan and Toffanin 1996). Rotation and correct allocation of crops may suffice to prevent economic damage to crops without the use of any specific control tool (Furlan et al. 2011).

Data from studies in Italy indicate that the most important factor in influencing wireworm population levels is crop rotation (Furlan and Talon 1997; Furlan et al. 2000), and this appears to be the situation in other regions (Eastern Europe, Hungary) as well (e.g. Szarukàn 1977). This is because meadows and the use of double cropping within the rotation cycle may result in population increases of a species that has the capacity to overwinter as adults (Furlan 2005). Therefore, any modification of these factors may disrupt wireworm population dynamics. Altering rotations, i.e. temporary removal of the most suitable crops for wireworm development, is a key agronomic strategy for population control.

Altering tillage timing, i.e. choosing a crop rotation that allows for soil tillage in the most critical phase of the wireworm life cycle (e.g. when most eggs are laid and the first instar larvae are in the soil), may also reduce wireworm populations (Furlan 1998, 2004). Tillage timing should be modulated in accordance with the life cycle differences among the main *Agriotes* species. Altering irrigation timing to ensure the drying of the topmost soil layer just after eggs are laid can also be an effective means of controlling *Agriotes* populations (Furlan 1998, 2004). Altering planting timing can also be effective, recognizing that a population’s capacity to damage sensitive plants varies with the season. For instance, even very high *A. ustulatus* populations do not damage maize because most of the larvae are in a non-feeding phase by late spring (Furlan 1998). Therefore, adjusting planting timing when possible to coincide with low pest populations or with non-damaging life stages can be effective. Another agronomic tool for population control is intercropping in which winter-wheat or other trap-crop plants are included in fields as a control strategy to draw pests away from the main economic crop (Furlan and Toffanin 1994; Vernon et al. 2000).

#### Applying biological tools for controlling wireworm populations

A range of other potential options are available for fields infested with damaging wireworm populations when planting the sensitive crop in non-infested fields has been ruled out (Furlan 2007). The mechanisms and effectiveness of some of these various control methods have been accurately assessed under controlled conditions (Furlan and Toffanin 1998; Furlan and Campagna 2002) and currently show that biocidal plants and seed meals are the only practical options (Furlan et al. 2009b, 2010). Their potential can be considered comparable to that of neonicotinoids and other chemical insecticides that can replace neonicotinoids (Ferro and Furlan 2012), especially when they are used to interfere with population development and not simply to reduce wireworm populations just before or during sowing (Furlan et al*.* 2009b, 2010).

#### Applying chemical insecticides for controlling wireworm populations

In fields where wireworm populations exceed economic thresholds and the agronomic and biological alternatives are not feasible, alternative insecticides to neonicotinoids, such as pyrethroids and phosphorganics, are available (Wilde et al. 2004; Ferro and Furlan 2012). They should be used sparingly, in accordance with best practices for pesticide applications. The effectiveness of the soil insecticides can be influenced by soil and weather conditions (e.g. heavy rain taking away insecticide active ingredient) that can result in protection failure for either neonicotinoids and their alternative insecticides (Ferro and Furlan 2012; Furlan et al. 2011, 2014). No significant differences in wireworm control between neonicotinoids and several alternative insecticides were reported by Wilde et al. (2004); trials in Italy conducted over a 10-year period suggest that the likelihood of failure is higher for some alternative insecticides (Ferro and Furlan 2012; Furlan et al. 2011, 2014).

### Controlling Western corn rootworm (*Diabrotica virgifera virgifera*)

Western corn rootworm (WCR) damage to maize in Europe is only a risk where continuous maize cropping is adopted, especially when cropping is prolonged for several years (Furlan et al. 2014; Kiss et al. 2005; Sivčev et al. 2009). However, economic damage only occurs in areas with high WCR populations. Where maize is rotated, WCR populations are usually held below the economically important threshold, and there is little risk of significant crop damage (Kiss et al. 2005; Meinke et al. 2009; Sivčev et al. 2009). Therefore, IPM for WCR should be based on a systematic rotation of crops and supported by information on pest development and population levels as stated by the Directive 2009/128/EC and confirmed by the Commission Recommendation 2014/63/EU (on measures to control *D. virgifera virgifera* Le Conte in Union where its presence is confirmed).

#### Accurate WCR population monitoring and damage prediction

Baited and non-baited traps are available to monitor WCR population levels (Schaub et al. 2011). The most widely used non-baited traps include yellow sticky traps, and they are readily available from various manufacturers. The most commonly used sticky trap for threshold assessment is Pherocon AM® (PhAM). Both USA and European authors have demonstrated that there is a correlation between the number of adults captured by yellow sticky traps (i.e. PhAM) and plant damage the following year (Blandino et al. 2014; Boriani 2006; Hein and Tollefson 1985; Kos et al. 2014). The US authors stated that economic thresholds would be exceeded when more than 40 beetles/PhAM trap/week (6 beetles/PhAM trap/day) were caught the previous year in one period (ca. 7 days) during the last 3 weeks of August (Hein and Tollefson 1985). In Italy, the threshold was 42 beetles/PhAM trap/day on average over a 6-week period after the beginning of adult flights (Boriani 2006; Blandino et al. 2014). In Croatia, the threshold was estimated at 41 adults/ PhAM trap in week 31 (Kos et al. 2014). Economic thresholds can greatly vary with climatic/agronomic conditions and prices of maize and insecticides (Oleson et al. 2005). Under low stress levels (suitable soil with sufficient water and nutrient supply), maize yield is not likely to be significantly reduced even with WCR population pressures causing a root damage score of 1 on the 0–3 scale (Oleson et al. 2005). In contrast, low root injury rates may cause yield reduction if high stress levels for maize cultivation occur (Oleson et al. 2005). In any case, the likelihood that a yield reduction occurs is negligible when WCR population pressure is very low (<0.3 root injury score on the 0–3 scale, Furlan et al. 2014). Based on trap monitoring network data, innovative statistical tools (De Luigi et al. 2011) can reliably identify or predict the areas where populations are high enough that they lead to reduced yield.

#### Agronomic strategies for controlling WCR populations

Although WCR arrived more than 6 years ago in southern Veneto (De Luigi et al. 2011), where rotation is dominant, population levels have remained low and economic damage has not been found, even in nearby continuous maize fields (Furlan et al. 2014). In areas of Veneto where crop rotation is not prevalent, average WCR population levels are high and the risk of root damage is considerable. Continuous maize may be rotated with any type of crop different from maize. Even Gramineae species that are closely related to maize may be used as a first or second crop after a winter crop (e.g. winter wheat + sorghum or ryegrass +sorghum). Maize itself may even be used as a second crop (e.g. winter wheat + maize) to interrupt a WCR cycle, provided that it is sown after the WCR eggs have hatched (Davis et al. 1996).

The aforementioned results suggest that a proper IPM approach would be to monitor long-standing continuous maize fields each year and when WCR population thresholds are exceeded, to rotate the maize with any other crop for only 1 year followed by monitoring in the subsequent maize crops. Periodic crop rotations disrupt the WCR life cycle, keep populations below economic thresholds, and typically preclude the need for insecticides. In practice, maize may be rotated at varying frequencies, even after several years of continuous maize cultivation, and only when monitoring reveals that WCR population levels are increasing. Crop rotations offer other agronomic benefits in addition to insect population management (Furlan et al. 2014; Saladini et al. 2009), thereby increasing incentives for periodic crop rotation.

The success of flexible rotation as an IPM strategy has also been confirmed by area-wide simulations (metamodels). These models have shown that 100 % rotation of maize is not necessary to keep regional WCR populations beneath economic thresholds, as, e.g. the interruption of continuous maize cropping after 3 years reduces the need for rotation to manage successfully WCR to below 60 % of the maize fields (Szalai et al. 2014). The use of variable rotation frequencies and crops may also be important where, such as was demonstrated in the USA, a “WCR variant” has adapted to crop rotations and are able to successfully lay economically significant levels of eggs outside of corn thereby causing damage to maize in a simple corn-soybean rotation (Levine et al. 2002).

In countries where allowed, another important agronomic alternative is transgenic corn that protects against WCR damage because the *Bacillus thuringiensis* protein expressed in the maize is toxic to WCR larvae (Meissle et al. 2011; Vaughn et al. 2005). Its efficacy has been shown to be better than neonicotinoid insecticides (Oleson and Tollefson 2005, 2006). This transgenic corn must be used under insect resistance management strategies (Onstad et al. 2001) and be integrated with other agronomic tactics to keep populations below the economic thresholds for “non transgenic” maize.

#### Applying biological tools for controlling WCR populations

Although rotation appears to be the most suitable measure for keeping WCR populations below economic thresholds, effective biological control options are also available as alternatives to chemical insecticides, with entomopathogenic nematodes proving to be a highly effective way of suppressing WCR populations under field conditions (Kurtz et al. 2007; Toepfer et al. 2010, 2013). Conversely, the parasitoid *Celatoria compressa* (Diptera: Tachinidae) does not appear to be viable for practical application at the moment (Toepfer and Kuhlmann 2004; Kuhlmann et al. 2005; Zhang et al. 2003).

#### Applying chemical insecticides for controlling WCR populations

Studies show that neonicotinoid seed treatments and soil applications used as in-furrow treatments at planting do not interfere significantly with WCR populations (Furlan et al. 2006). In situations where an IPM process is still insufficient to control crop damage and some maize fields require insecticide protection, alternative insecticides to neonicotinoids are available. For example, pyrethroids and phosphorganics can be as effective as neonicotinoids against WCR (Agosti et al. 2011; AA.VV. 2012; Blandino et al. 2013; Furlan et al. 2006; Waldron et al. 2002; Whitworth and Davis 2008) or even more effective (Oleson 2003; Oleson and Tollefson 2005). Protection against WCR by insecticides is less effective than protection by crop rotation, and insecticide effectiveness can be influenced by soil and weather conditions and by WCR population pressure that can result in protection failure (Boriani 2008, Furlan unpublished data).

Foliage insecticide treatments (e.g. with pyrethroids and phosphorganics) against WCR beetles may sometimes (i) protect maize silks from beetle chewing if applied before flowering, but this is needed only with very high WCR populations (Furlan, unpublished data) that should not be the case when IPM strategies are implemented; and (ii) actually reduce WCR population levels and the subsequent oviposition by females. The use of a development model (Nowatzki et al. 2002) may help to identify the period in which foliage insecticide treatments can significantly reduce the oviposition of females. Furthermore, this development model indicates whether treatment to contain corn borers (e.g. *Ostrinia nubilalis*) would also reduce WCR adult numbers leading to non-economic population levels in the following year. However, foliage treatments should be used with caution and only when other options under an IPM approach have not been successful or are not feasible because wide scale use of insecticides can lead to (i) resistance as already demonstrated in WCR larvae (Ball and Weekman 1962) and adult beetles (Meinke et al. 1998), (ii) outbreaks of secondary pests such as red mites, and (iii) possible environmental impacts.

Based on the principles of IPM and the evidence from numerous field trials in Italy described above, there is strong evidence that neonicotinoids are not required for effective management of WCR damage in maize. These principles and alternatives have also been successfully applied in the USA under an Area-Wide Pest Management scheme for rootworm control in corn fields (French et al. 2007).

### Controlling black cutworm (*Agrotis ipsilon*)

The majority of attacks on maize in Northern Italy are caused by an invasive species, the black cutworm (BCW) *A. ipsilon* Hufnagel (Furlan et al. 2001c). This species normally cannot overwinter in the conditions of Northern Italy and other northern regions (Zangheri et al. 1998), but rather, outbreaks are due to invasions by massive flights from southerly areas. Insecticide applications at the time of sowing are not recommended because BCW cannot be detected at the time of sowing and because many insecticides applied at planting become less effective over time, whereas outbreaks often occur many days after sowing (Furlan et al. 2001c; Zangheri and Ciampolini 1971; Zangheri et al. 1984) resulting in insufficient control (Furlan 1989; Shaw et al. 1998). However, it has been shown in the USA that rescue treatments (post-emergence applications) using non-neonicotinoid insecticides can be very effective (close to 100 % control, Shaw et al. 1998).

An IPM approach to managing BCW is based on a combination of large-scale pheromone trap monitoring to detect population levels, the analysis of southerly winds that may carry flying moths, and a development model (Black Cutworm Alert programme, Furlan et al. 2001c; Showers 1997). More intensive local-level population monitoring (e.g. scouting of farm fields) is performed only when area-wide monitoring has established that there is a risk. When trap monitoring and wind analysis have established whether and where any moths are present, the degree-day accumulation is calculated, preferably with soil temperature (each day: (maximum temperature − minimum temperature)/2 − 10.4 °C developmental threshold temperature, Luckmann et al. 1976). Once the predicted risk date is reached (176°-day accumulation when the fourth larval instar forms in the fields), at-risk areas should be monitored for BCW larvae so that appropriate reduced risk insecticides can be used post-emergence, should the average amount of affected crops exceed the 5 % threshold. This reduces the overall amount of insecticide required, and this approach has been tested and demonstrated to be successful in USA and Italy for several years (Furlan et al. 2001c; Showers 1997).

There is evidence that some transgenic maize hybrids can potentially protect against BCW because the *B. thuringiensis* protein expressed in the maize is toxic to BCW, but this may not be as effective as rescue treatments with appropriate insecticides (Kullik et al. 2011). In addition, the use of transgenic corn for BCW control, as it was suggested for WCR control, has to be decided when it is not possible to know if a BCW economic threshold population is actually present or developing. This constraint makes the transgenic corn option of limited use in an IPM approach against BCW.

We suggest that the IPM strategies for major insect pests that we illustrate in a European maize production system can be applicable to maize production in other countries as well, with some adaptations where other minor pests are present. The overall process for the three major pests we discuss can be summarized as follows: no prophylactic chemical treatments at maize sowing, black cutworm control where and if thresholds are exceeded based on Black Cutworm Alert programme supplemented by scouting when and where needed, WCR kept under control mainly by agronomic strategies, and treatments against wireworms restricted to the minor part of fields with populations exceeding the thresholds detected with the monitoring procedure described above. The cost and crop damage risk of an IPM approach can be effectively minimized by a mutual fund system (a special type of crop insurance directly managed by farmer associations) that ensures a guaranteed farm income in all cases.

## Case study of alternative pest management in Canadian forests

The emerald ash borer, *Agrilus planipennis* (Coleoptera: Buprestidae), is a wood-boring exotic invasive insect pest that is increasingly threatening the health and survival of ash (*Fraxinus* spp.) trees in large regions of eastern North America (Poland and McCullough 2006; McCullough and Siegert 2007). All North American ash species are susceptible to emerald ash borer, and mortality of ash trees occurs rapidly after infestation. Ash is an important urban forest species, but it can also dominate in landscapes associated with water, such as riparian (shoreline) buffers along agricultural runoff streams and ravines, temporary pools and wetlands, and in headwater or source water areas. In this regard, ash can be a keystone forest species that influences or regulates riparian forest and aquatic ecosystem dynamics and nutrient cycling through canopy cover and leaf litter inputs to forest floors and water bodies (Ellison et al. 2005; Gandhi and Herms 2010; Flower et al. 2013). Therefore, the rapid loss of ash from these ecologically sensitive areas can pose a risk to critical habitats, biodiversity, and some important ecosystem services.

As a first step toward managing the damage from emerald ash borer when the pest populations begin to build, three management options have been proposed to slow the spread and infestation by the insect. These are (i) cutting and removing living ash trees in advance of the infestation, (ii) girdling living ash trees on the leading edge of an infestation, and (iii) the application of an effective systemic insecticide (McCullough and Poland 2010; Mercader et al. 2011). Intentionally removing some of the living ash trees before or in early stages of the infestation reduces the phloem available for larval development. This approach also provides opportunities for forest canopy redevelopment by other tree species through natural regeneration or strategic under-planting to minimize impacts from the sudden loss of ash by the emerald ash borer infestation (Streit et al. 2012). Girdling living ash trees on the leading edge of an infestation causes the stressed tree to act as a trap tree to which egg-laying females are attracted in large numbers, presumably because of increased attractive volatiles and/or visual cues (McCullough et al. 2009). Those trap trees are then destroyed before larval development, thereby concentrating the future cohort of the emerald ash borer to a specific area and reducing the local population.

The third management option to reduce tree mortality and slow the spread of emerald ash borer is the application of a systemic insecticide. A systemic insecticide is well suited for control of this insect pest because the damaging life stage of the pest is the phloem-feeding larvae. Among the systemic insecticides that have been shown to be effective against the emerald ash borer is the neonicotinoid, imidacloprid (Poland et al. 2006). Applications to trees can be made by soil injections around the base of individual trees or by direct stem injections into tree trunks. However, Canadian field and laboratory studies showed that autumn-shed leaves from imidacloprid-treated trees can contain residues that pose risk of harm to aquatic and terrestrial decomposer organisms through sublethal feeding-inhibition effects (Kreutzweiser et al. 2007, 2008a, 2009). They further showed that field-realistic concentrations of imidacloprid in soils and water posed direct risk of adverse effects to earthworms (Kreutzweiser et al. 2008b) and aquatic invertebrates (Kreutzweiser et al. 2008c). These results, coupled with a commitment to adopt an IPM approach to the emerald ash borer problem, prompted an examination of alternatives to imidacloprid for emerald ash borer control.

In a forest insect pest context, an IPM approach examines and applies a combination of management methods using all available information to make informed management decisions. This approach currently being applied to the control of emerald ash borer in Canada includes studies into the pest biology and behaviour to facilitate biological control (Lelito et al. 2013), effective and practical traps for the highly mobile adults to track infestations (Grant et al. 2010; Ryall et al. 2013), improved detection methods for locating early infestations and potential hot spots (Ryall et al. 2011), and alternative pest management strategies. Here, we briefly describe some of the alternatives to imidacloprid being explored for the control of emerald ash borer in Canada.

### Exotic parasitic insects

Three species of hymenopterous parasitoids (parasitic wasps) were found to parasitize emerald ash borer larvae or eggs in China, and these are being reared in the USA as potential biological control agents (Lyons 2013). The emphasis on finding, importing, and rearing exotic parasitoids was on selecting species that show a high degree of host specificity. The three species, Braconidae: *Spathius agrili*, Eulophidae: *Tetrastichus* *planipennisi*, and Encyrtidae: *Oobius agrili*, have been released annually since 2007 in northeastern USA under biological control regulations (Gould et al. 2012) and their populations are being monitored. Early indications are that at least one species (*T. planipennisi*) has been successful in establishing a measureable population and has the potential for beginning to control emerald ash borer infestations (Duan et al. 2013). *T. planipennisi* was released at two sites in Canada in 2012 and monitoring is ongoing to determine the success of population establishment (B. Lyons, personal communication).

### Native parasitic insects

Surveys were conducted in emerald ash borer-infested areas of Canada to determine if native parasitoids were active on, or associated with, the invasive insect pest. Several species of hymenopterous parasitoids were encountered in these surveys and were trapped and reared to determine a parasitism rate for each species on emerald ash borer. Among those, only a few (e.g. Chalcididae: *Phasgonophora sulcata*, Braconidae: *Atanycolus hicoriae*) have shown relatively high rates of parasitism on emerald ash borer and hold some promise as a native biological control agent (Lyons 2010). Efforts are ongoing to determine the potential for native parasitoids to assist biological control strategies using parasitic wasps. This includes developing techniques for rearing and releasing or otherwise augmenting natural populations of promising native parasitoids. The combined use of exotic and native parasitoids as biocontrol agents may eventually be successful in helping to manage emerald ash borer populations, but they are still in the early stages of development.

### Native fungal pathogens

The use of native entomopathogenic fungi as biological control agents against emerald ash borer is being explored in Canada. Screening of prepupal and adult cadavers from established emerald ash borer populations indicated that the most prominent natural pathogenic fungus on emerald ash borer was *Beauveria* spp. (Kyei-Poku and Johny 2013). These were subsequently isolated and characterized, and it was determined that the L49-1AA isolate of *Beauveria bassiana* was the most promising in terms of virulence against emerald ash borer (Johny et al. 2012). An effective entomopathogenic fungus requires an efficient dissemination system to spread the fungus among susceptible hosts of the pest population. Lyons et al. (2012) developed an autocontamination trap system for emerald ash borer in which adults are contaminated with *B. bassiana*, and they found evidence that this system facilitated horizontal transmission among adults.

Entomopathogens show some promise as biological control agents and some methods for their screening, characterization, and dissemination have been developed. However, there are still some limitations of this approach for broad-scale control of emerald ash borer. Entomopathogens in general do not appear to be significant factors that regulate emerald ash borer populations (Liu et al. 2003), and the pest’s biology and behaviour do not lend themselves to efficient fungal transmission. Moreover, many entomopathogens, including *B. bassiana*, are not particularly host-specific, and if they are disseminated as biological control agents, they may pose risks to non-target insects.

### An alternative, non-persistent systemic insecticide

Several systemic insecticides were screened for efficacy against emerald ash borer, their translocation efficiencies in ash trees, and their environmental safety. The most promising of these was azadirachtin. Azadirachtin is a natural compound extracted from the seeds of the neem tree, *Azadirachta indica*, and has been shown to have antifeedant, antifertility, and growth-regulating insecticidal properties against a range of insect pests (Schmutterer 1990). Previous studies in a Canadian forestry context showed that azadirachtin was not persistent in the environment (water, soils, tree foliage) and did not present significant risk to most non-target invertebrates at expected environmental concentrations (Thompson and Kreutzweiser 2007), and therefore, it was considered a strong candidate for control of emerald ash borer. Azadirachtin was injected into trunks of infested ash trees and shown to be highly effective at inhibiting larval development and adult emergence and, therefore, effective in protecting ash trees from the wood borer (McKenzie et al. 2010). Subsequent field trials confirmed that azadirachtin is readily taken up following stem injection of ash trees, is rapidly translocated throughout the tree and to foliage, and usually dissipates to near limits of detection in autumn-shed leaves (Grimalt et al. 2011). We conducted a suite of non-target tests following protocols of those used to assess the effects of imidacloprid and showed that azadirachtin in autumn-shed leaves poses no measurable risk of harm to terrestrial or aquatic decomposer invertebrates, even after intentionally high application rates (Kreutzweiser et al. 2011).

## Conclusions

These case studies in agriculture and forestry provide examples of reasonable and viable alternatives to neonicotinoid insecticides for control of insect pests. In the agricultural setting, it is becoming increasingly clear that prophylactic insecticide treatments with neonicotinoids are often not needed and result in unnecessary contamination of the environment thereby increasing risks to non-target organisms (van der Sluijs et al. 2014) and may increase the likelihood of developing resistance among insect pests (Szendrei et al. 2012). As an alternative, an IPM approach should consider all relevant and available information to make informed management decisions, providing pest control options based on actual need. When a need is identified, pest control options that preclude the use of neonicotinoid insecticides are varied and may include diversifying and altering crop rotations, planting dates, tillage, and irrigation; using less sensitive crop species in infested areas; applying biological control agents; and turning to alternative reduced risk insecticides. These options are often most effective when applied in combination under an overall IPM strategy.

Widespread adoption of an IPM approach to insect pest management will require education and acceptance by regulators and practitioners. As an example, a particularly promising incentive for IPM implementation in Italy is a yield insurance scheme (mutual fund) for farmers, in which the required insurance premium is usually lower than insecticide costs (Furlan et al. 2014). An initial public contribution to this kind of crop insurance scheme to offset the risks of IPM implementation would encourage wider adoption of IPM strategies.

We recognize that the adoption of alternatives to neonicotinoids and moving agricultural practices to an IPM approach is particularly challenging where large-scale, cost-effective agricultural operations are on the landscape. Over the past two decades, the trend toward large, commercial agricultural operations has focused on scale economies and efficiencies (Morrison Paul et al. 2004), and this has encouraged the use of prophylactic crop protection by neonicotinoids to reduce risks from pests. Shifting agricultural production from a reliance on prophylactic insecticides to an IPM model and the use of alternative pest control options will take some time and will require investments in research and public extension to promote economically competitive and sustainable agricultural systems (Meissle et al. 2010). However, staying the course of widespread and prophylactic use of neonicotinoids increases the risk of serious environmental harm (van der Sluijs et al. 2014) and may ultimately threaten important ecosystem functions and services that support food security (Chagnon et al. 2014). Implementing sustainable agricultural practices at regional scales would benefit from a landscape perspective and the adoption of landscape design principles based on incentives or regulations (Dale et al. 2013).

While some of the options for alternative pest control that we illustrate in these case studies have been successfully demonstrated and field-tested, others are under ongoing development. Continued research into alternatives is warranted, but equally pressing is the need for transfer and training of IPM technologies for farmers and other practitioners by public agencies and the need for policies and regulations to encourage the adoption of IPM strategies and their alternative pest control options.
